# TRAF Regulation of IL-17 Cytokine Signaling

**DOI:** 10.3389/fimmu.2019.01293

**Published:** 2019-06-27

**Authors:** Shadi Swaidani, Caini Liu, Junjie Zhao, Katarzyna Bulek, Xiaoxia Li

**Affiliations:** ^1^Department of Inflammation and Immunity, Cleveland Clinic, Lerner Research Institute, Cleveland, OH, United States; ^2^Department of Cardiovascular & Metabolic Sciences, Cleveland Clinic, Lerner Research Institute, Cleveland, OH, United States

**Keywords:** IL-17, TRAF, inflammation, signaling/signaling pathways, Act1

## Abstract

Tumor necrosis factor receptor (TNFR)-associated factors or (TRAFs) are important mediators of Interleukin-17 (IL-17) cytokine signaling and contribute to driving tissue responses that are crucial for protective immunity but are often implicated in immunopathology. By amplifying tissue immune activity, IL-17 cytokine pathways contribute to maintaining barrier function as well as activation of innate and adaptive immunity necessary for host defense. IL-17 receptors signaling is orchestrated in part, by the engagement of TRAFs and the subsequent unlocking of downstream cellular machinery that can promote pathogen clearance or contribute to immune dysregulation, chronic inflammation, and disease. Originally identified as signaling adaptors for TNFR superfamily, TRAF proteins can mediate the signaling of a variety of intercellular and extracellular stimuli and have been shown to regulate the downstream activity of many cytokine receptors including receptors for IL-1β, IL-2, IL-6, IL-17, IL-18, IL-33, type I IFNs, type III IFNs, GM-CSF, M-CSF, and TGF-β Toll-like receptors (TLRs), NOD-like receptors (NLRs), RIG-I- like receptors, and C-type lectin receptors. This review will focus on discussing studies that reveal our current understanding of how TRAFs mediate and regulate biochemical activities downstream of the IL-17 cytokines signaling.

## TRAF Molecules

Discovered in 1994, Tumor necrosis factor receptor (TNFR)-associated factors or TRAFs are a phylogenetically conserved and structurally related family of cytoplasmic proteins composed of seven known members TRAF1 through TRAF7. While TRAFs were initially implicated in the regulation of inflammatory responses through their engagement with TNFR2 (1), they are now recognized to engage diverse signaling cascades functioning as central regulators of immunity and inflammation. All identified mammalian TRAF molecules, with the exception of TRAF7 (2, 3), constitute the hallmark highly conserved carboxyl terminal TRAF domain (TRAF-C) through which they are recognized as intracellular scaffolding molecules. TRAF molecules can engage cytoplasmic domains of cytokine receptors i.e., IL-1β, IL-2, IL-6, IL-17, IL-18, IL-33, type I IFNs, type III IFNs, GM-CSF, M-CSF, and TGF-β, Toll-like receptors (TLRs), NOD-like receptors (NLRs), RIG-I- like receptors, and C-type lectin receptors (4). TRAFs self-assemble into trimeric homo or heterotrimers of TRAF-C domains mediated in part by the amino terminal extension of the TRAF-C domain that contains a coiled-coil domain. TRAF2 coiled-coil domain is the longest and contains 14 heptads, compared to only 3 heptads on TRAF4 (5).

In addition, with the exception of TRAF1, all TRAFs also contain an amino terminal (really interesting new gene) or RING finger domain endowed with E3 ubiquitin ligase activity thought to be necessary for TRAF function (6). E3 ubiquitin ligase activity of the RING domain has been shown to be required for TRAF-dependent activation of nuclear factor kappa-B NFκB1 and NFκB2, mitogen-activated protein kinases (MAPKs); extra-cellular signal-regulated kinase (ERK), P38, and Jun N-terminal protein kinase (JNK) as well as interferon regulatory factors/IRFs and downstream gene expression (7). Biochemical studies also suggests that TRAFs are capable of mediating Lysine 29(K29), lysine 48(K48), lysine 63(K63) polyubiquitination, the later, shown to require RING domain dimers and the most proximal zinc finger to RING, zinc finger 1 (ZF1) (8–10). Interestingly, It was recently demonstrated that TRAFs i.e., TRAF6 molecules can form trimers that could associate to enable the formation homo or heterodimer RING assemblies with TRAF5 (11). It seems TRAF6 RING dimers are important for the formation of a catalytic complex with the ubiquitin conjugating E2 enzyme and E3 ligase activity. It is proposed that, at least for TRAF6 dimers which utilizes Ubc13-Uev1A E2 complex (12), one RING interfaces with a Ubc13~Ubiquitin conjugate, while the zinc finger 1 (ZF1) domain and linker-helix of the opposing monomer associates with ubiquitin (11).

## IL-17 Cytokine Family

Interleukin-17(IL-17) cytokine family members are known to promote and drive tissue responses that are crucial for protective immunity. IL-17 cytokines are also implicated in mediating acute and chronic inflammation, autoimmunity, and cancer. Homology-based cloning has revealed six IL-17 family members, termed IL-17A, IL-17B, IL-17C, IL-17D, IL-17E, and IL-17F (13). IL-17A commonly referred to as IL-17, is the prototypical IL-17 family member and plays an essential role in host defense against microbial infections and is widely implicated in various inflammatory conditions i.e., psoriasis, asthma, rheumatoid arthritis, multiple sclerosis, transplant rejection, inflammatory bowel disease, and cancer (14, 15). IL-17A is produced by type 17 T helper (Th17) cells, B cells, γδT cells, iNKT cells, a subset of CD8+ T cells, known as Tc17 cells, type 3 innate lymphoid cells (ILC3s) (16), as well as a subset of human regulatory T cells(17). The transcription factor retinoid-related orphan nuclear receptor γt (RORγt) is required for the generation and propagation of IL-17 producing type 17 T helper cells (18). Interestingly, TRAF5 mediates Lysine 63(K63) polyubiquitination and stabilization of RORγt and can promote IL-17 production and pathology.

IL-17A amplifies tissue immune activity by promoting the production inflammatory mediators; cytokines, chemokines, acute phase proteins, anti-microbial peptides, and matrix metalloproteinases. Further, can drive granulopoiesis and tissue neutrophilia; as well as maintaining barrier function by the activation of innate and adaptive immunity necessary for host defense. Dysregulation of IL-17 activity under pathological conditions is implicated in leading to chronic inflammation, tissue damage, and disease.

Biological responsiveness to IL-17 cytokines is mediated by five single pass transmembrane IL-17 receptors (IL-17RA, IL-17RB, IL-17RC, IL-17RD, IL-17RE) that possess the hallmark cytoplasmic SEFIR domain (similar expression of fibroblast growth factor and IL-17R), a motif uncovered through phylogenic sequence analysis to be similar to the Toll/IL-1R (TIR) domain found in IL-1 and Toll-like receptor family members and comprising the STIR superfamily (19). While debate still exists regarding the stoichiometry of the IL-17 receptor complex, biochemical, genomic, and structure function analysis suggests that the SEFIR domain mediates both homotypic and heterotypic interactions among IL-17Rs and its adaptor molecule Act1 (20). The SEFIR domain is also present on Act1 (NFκB activator 1, also known as CIKS or TRAF3IP2) which was originally discovered as an activator of NFκB pathway. Act1 is now known to be recruited to the IL-17 receptor complex in a manner dependent on the homotypic and heterotypic interactions between the SEFIR domains on Act1 as well as IL-17 receptors (21). IL-17 receptor complex also elicits a dual phosphorylation of the CAAT enhancer binding protein β (C/EBPβ) at Threonine 188(Thr188) and Threonine 179(Thr179) that is mediated by ERK and glycogen synthase kinase 3β (GSK3β) (22). The distal domain of IL-17RA -coined C/EBPb-activating domain (C-BAD)- is necessary for phosphorylation of C/EBPβ and downstream inhibition of IL-17-dependent gene induction. It is also suggested that during chronic activation of IL-17 pathway, stimulates Lys48-linked polyubiquitination and degradation of IL-17 receptor adaptor; Act1 (23).

Considerable sequence divergence exists between IL-17R genes, which are located on human chromosome 22 (IL-17RA) and human chromosome 3 (IL-17RB, IL-17RC, IL-17RD, IL-17RE) (24, 25). A sixth member of the IL-17 receptor family was identified as IL-17REL (26). IL-17REL was found to be associated with inflammatory bowel disease, ulcerative colitis (27), and gout (28); yet, its biological role remains poorly defined. IL-17 Cytokines require a heterodimer receptor complex that must include the common IL-17 receptor chain IL17RA in addition to the supplementary chain IL-17RC for hematopoietic cell derived IL-17A and epithelial derived IL-17F, IL-17RB for epithelial and hematopoietic derived IL-17E (IL-25) and epithelial IL-17B, and IL-17RE for epithelial derived IL-17C (29).

Despite the obligate requirement for IL-17RA chain for IL-17A, IL-17B, IL-17C, IL-17D, as well as IL-17E (IL-25), IL-17 cytokines can induce distinct biological responses. While much overlap is observed in downstream target gene in IL-17A; IL-17C, and IL-17F; the cellular sources and co-receptor are thought to have different expression and tissue localization.

In comparison to IL-17A, IL-17E or IL-25 is often deemed the black sheep of the group. IL-17E or (IL-25) signaling shares some similarities with IL-17A signaling transduction but has very defined biological effects and is linked to allergic, atopic inflammation, and eosinophilic responses. IL-25 signals through a heterodimeric receptor complex IL-25R which also utilizes the common IL-17RA and addition to IL-17RB, both of which contain a conserved SEFIR domain at the cytoplasmic region. Upon binding of the IL-17E (IL-25), IL-25R recruits the adaptor molecule Act1 through the homotypic and heterotypic interactions of the SEFIR domains (30, 31). Act1 deficiency results in a loss of IL-25-dependent gene expression and renders mice resistant to IL-25-mediated allergic airway inflammation.

## TRAF Regulation of IL-17 Cytokine Signaling

### TRAF6

TRAF6 is ubiquitously expressed in human tissues and is highly expressed in bone marrow. TRAF6 is involved in both innate and adaptive immune pathways through the signaling of several cytokines and intracellular mediators. TRAF6 is shown to engage TIR domain containing cytokine receptors of IL-1R family; IL-1R (32), IL-18R, and IL-33R (33, 34), MyD88-dependent TLRs, NLR family; NOD1, NOD2 (35), and RIG-I/RLRs (36). TRAF6 is also required for the activation of both canonical and non-canonical NFκB pathways as well as activation of the MAPKs (JNK1/2, ERK1/2, and p38) and IRFs (IRF3, IRF4, IRF5, and IRF7).

**Figure 1:**
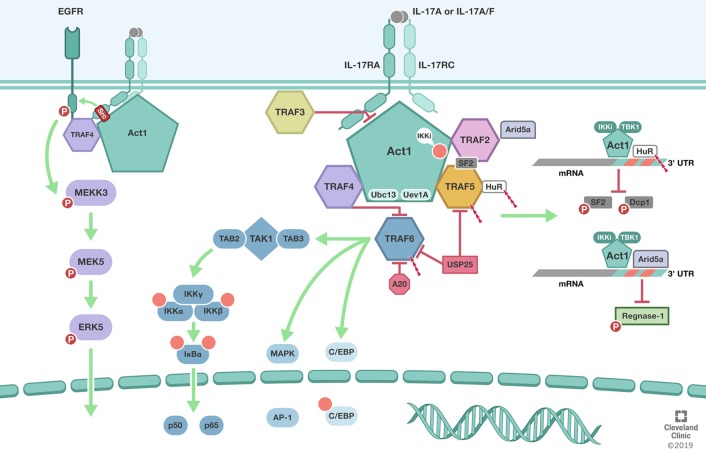
TRAFs in IL-17 signaling pathway. Biological responsiveness to IL-17 is mediated by single pass transmembrane IL-17 receptors (IL-17RA, IL-17RC). **ACT1** U-box E3 ubiquitin ligase function in conjunction with the ubiquitin conjugating enzyme Ubc13 and Ubc-like protein Uev1A mediates K63-linked polyubiquitination of **TRAF6**. TRAF6 mediated deubiquitination by USP25 and A20 mediate the negative feedback regulation of IL-17 induced NFκB and MAPK. In addition to its role as the adaptor molecule for IL-17 receptors, Act1 also functions as an RNA-binding protein that mediates receptor-induced stabilization of select mRNAs by directing the formation of distinct RNA-protein complexes. Act1-**TRAF2-TRAF5** complex modulation of mRNA stability is dependent on IkB kinase (IKKi) and TBK1-mediated phosphorylation of Act1 at the serine residue 311 (Ser311). Formation of Act1/TRAF2/TRAF5/ASF (arginine serine rich splicing factor) also known as SRSF1 or SF2 prevents ASF from binding to the 3′ UTR of CXCL1 mRNA and subsequent degradation. **TRAF2** also recruits IL-17-induced RNA binding protein Arid5a to stabilize mRNA by counteracting mRNA degradation mediated by the endoribonuclease Regnase-1. **TRAF3** can bind directly to the IL-17R to impede the formation of the IL-17R-Act1-TRAF6 complex. **TRAF4** tethers IL-17RA and EGFR to enable IL-17A-induced EGFR transactivation and downstream activation of ERK5. In addition, **TRAF4** competes with TRAF6 for the two TRAF binding site (TB) on Act1. Illustration by Shadi Swaidani MD, Ph.D. and Brandon Stelter, BFA, Reprinted with the permission of the Cleveland Clinic Center for Medical Art & Photography © 2019. All Rights Reserved.

TRAFs were initially implicated in the IL-17 signaling pathway by the reported requirement of TRAF6 but not TRAF2 in IL-17 mediated NFκB pathway activation (37). It was subsequently shown that the cytosolic protein Act1, is crucial for their biochemical interaction and subsequent TRAF6 mediated NFκB activation and downstream gene expression. ACT1 U-box E3 ubiquitin ligase function in conjunction with the ubiquitin conjugating enzyme Ubc13 and Ubc-like protein Uev1A mediates K63-linked polyubiquitination of TRAF6 (38). Act1 mediated TRAF6 recruitment and polyubiquitination primarily on Lysine 124(K124) is required for TRAF6-dependent TGFβ-activated kinase 1(TAK1) TAK1-IKK [composed of IKKα, IKKβ, and IKKγ/NEMO] and IKK mediated IkB**α** phosphorylation and proteasomal degradation. The later allowing the nuclear localization p50-p65 dimers and the activation of the classical (or canonical) NFκB signaling pathway. Interestingly, the non-receptor tyrosine kinase Spleen tyrosine kinase (Syk) was also shown to participate in the Act1/TRAF6 complex and to be necessary for K63-linked polyubiquitination of TRAF6, IL-17A-induced TAK1, IKK, IkBα, JNK, and MKK4 and chemokine ligand 20 CCL20 mRNA and protein levels (39). Interference with Syk expression reduced Act1 interaction with TRAF6 and attenuated NFκB activation and downstream CCL20 levels (Figure 1).

TRAF6 was also crucial for IL-25R-mediated NFkB activation and IL-25 induced gene expression of IL-6, TGFβ, G-CSF, TARC (Thymus and activation-Regulated chemokine) but not activation of MAPK pathway (40). These studies demonstrated that IL-25 induced activation of ERK, JNK, P38 was intact in the absence of TRAF6, suggesting that MAPK downstream pathway is independent from TRAF6 unlike IL-25R-mediated NFkB activation and gene expression. Although, IL-17RB-SEFIR which contains a putative TRAF binding motif does not function as a true TRAF6-binding motif, it is possible that IL-17RB interaction with TRAF6 directly via a still undefined motif (41). In addition, IL-17RB possibly recruits TRAF6 indirectly via the heterodimeric interaction with IL-17RA and the adaptor protein Act1.

Noteworthy was the identification that IL-17RD engages TRAF6 and is part of the heterotrimeric complex required for IL-17 signaling and gene expression (42). IL-17RD was found to disrupt IL-17RA interaction with Act1 and TRAF6 (43). IL-17RD was implicated in down regulating the NFκB pathway while promoting p38 MAPK pathway.

Thus, TRAF6 is a necessary component of the positive induction of downstream signaling of both IL-17A as well as IL-17E/IL-25. TRAF6 is recruited to IL-17RA, IL-17RB, and IL-17RC by Act1. TRAF6 recruitment to the receptor complex and K63 polyubiquitination primarily on Lysine 124(K124) is required for induction of the pathway. Conversely, negative regulation of this pathway is also mediated in part by TRAF6. USP25 and A20 (44) both function as deubiquitinase enzymes or DUBs that target K63-linked polyubiquitination of TRAF6 and mediate the negative feedback regulation of IL-17 induced NFkB and MAPK.

### TRAF2/5

While TRAF2 and TRAF5 are implicated in cytokine mediated activation of the both classical (canonical) and alternative (non-cononical) NFkB pathways, MAPKs, IRFs and can contribute inflammasome activation during chronic inflammatory responses (45–47), their mechanistic involvement in the IL-17A signaling pathway has been shown to potentiate distinct effects. IL-17R signaling through Act1-TRAF6 interaction has been shown to activate the classical NFkB pathway and downstream IL-17 target genes, albeit, less robustly than potent proinflammatory cytokines i.e., TNFα, and IL-1β; IL-17A has been shown to amplify TNFα-stimulated mRNAs in wild-type, as well as TRAF6-deficient cells, but not from Act1 deficient cells (48). IL-17A capability to stabilize TNFα induced KC, MIP-2, and IκB**ζ** mRNAs in TRAF6-deficient cells remains intact. These observations were suggestive of the independence of the classical NFkB pathway from the mRNA stability mechanism despite Act1 being central to both. IL-17A signaling through Act1-TRAF2-TRAF5 complex results in the control of mRNA stability of IL-17 as well as additional proinflammatory cytokines TNFα, and IL-1β target genes. This mRNA stabilization pathway is dependent on IkB kinase (IKKi) and TBK1-mediated phosphorylation of Act1 at the serine residue 311 (Ser311) (49, 50). Phosphorylation of Act1 at Ser311 allows the recruitment and interaction of TRAF2 and TRAF5, but not TRAF6, to form an Act1/TRAF2/TRAF5/ASF (Arginine Serine rich splicing factor) also known as, SRSF1 or SF2. Formation of this complex prevents ASF from binding to the 3′ untranslated regions UTR of CXCL1 mRNA and subsequent degradation.

Evidently, through Act1-TRAF2/5 dependent mechanism, IL-17A signaling can also activate the mRNA stabilizing factor human antigen R (HuR)/ELAVL1 (51). IL-17A induced mRNAs exhibit short half-life's due to adenine and uridine–rich sequence elements (AREs) within their 3′ (UTRs) that can mediate deadenylation, decapping, and exonucleolytic degradation by ARE-binding proteins. IL-17A stimulation induced Act1-dependent, K63-linked polyubiquitination of HuR and interaction of HuR with Act1 and TRAF2/5 but not SF2/ASF. HuR may compete with ASF for binding to the ARE-mRNAs i.e., CXCL1 and CXCL5 and promote mRNA stability and enhance neutrophilic inflammation. Interestingly, IKKi deficiency does not impact the IL-17-Act1-TRAF6 axis and the activation of NFkB but instead leads to an attenuated activation of (MAPKs) and attenuates IL-17-dependent stabilization of mRNA encoding the neutrophil chemokine CXCL1 and tissue neutrophilic infiltration. While ASF and HuR are implicated in IL-17-induced mRNA regulation, the recruitment of ASF and HuR cannot explain the receptor-specific mRNA stabilization of selected IL-17 inflammatory genes. Unexpectedly, while Act1 is the essential adaptor molecule for IL-17 receptors, recent study has shown that it also exhibits direct mRNA-binding activity. Importantly, much of the receptor-mediated target specificity is mediated by Act1 and its RNA-binding capacity that allows it to interact with the stem-loop structure of select inflammatory mRNAs. A more detailed discussion of this emerging aspect of IL-17-mediated mRNA stabilization can be found in recent publications (52, 53).

The positive role of TRAF2/5 in IL-17A signaling is regulated by ubiquitinylation. It has been shown that ubiquitin-specific protease 25 (USP25) deubiquitinates Act1-mediated K63 polyubiquitination of both TRAF5 and TRAF6, turning down IL-17A mediated mRNA stability and NFkB pathways, respectively (54). Notably, a recent study has shown that IL-17A stimulation induces the RNA binding protein Arid5a, which is recruited by TRAF2 to counteract Regnase-1-mediated mRNA degradation (53). Of importance, Regnase-1 can also be phosphorylated by IKKi and TBK1 in an Act1-dependent manner, which releases Reganse-1 from endoplasmic reticulum to preserve mRNA for translation (55).

While it was demonstrated that neither TRAF2 nor TRAF5 can associate with IL-17RB (40), TRAF2 was shown to interact with IL-17RD through TNFα induced activation and TNFR2. IL-17RD and TNFR2 complex formation was induced with TNFα and activated NFκB (56). Thus, through a unique mechanism, TRAF2 can multitask TNFα signaling through the engagement of IL-17 receptors namely, IL-17RD.

### TRAF3

TRAF3 is also ubiquitously expressed and plays important functions in regulation of antiviral immunity, inflammation, and tumor suppression (2, 4). Dysregulation of TRAF3 has been shown to promote autoimmunity and predisposition to cancer TRAF3 is an important negative regulator in TNF family receptors like CD40, B cell–activating factor (BAFF) receptor, and lymphotoxinβ receptor and has been shown to be required for Toll-like receptors (TLRs), RIG-I-mediated type I IFN production for antiviral defense and anti-inflammatory mediators i.e., IL-10 (57). Mechanistically, in the absence of ligand stimulation, TRAF3 requires TRAF2 for its activity (58). NFkB– inducing kinase (NIK) binds to TRAF3 and recruits TRAF2-cIAPs that mediate K48-linked ubiquitination of NIK and consequential proteasomal degradation. Upon ligand-receptor binding, TRAF3 is recruited to receptor complex and competes with NIK receptor interaction leading to its release accumulates and subsequent activation of the non-canonical NF-κB. Important to note that despite TRAF3 shared homology with TRAF2, they both poses non-redundant functions. In a similar fashion, TRAF3 mediates negative regulation of the IL-17 signaling. TRAF3 can bind directly to the IL-17R to impede the formation of the IL-17R-Act1-TRAF6 complex. IL-17 stimulation induces the recruitment of TRAF3 to the distal domain of IL-17RA and has been shown to compete with Act1 interaction. Over-expression of TRAF3 suppressed IL-17–induced IkBα phosphorylation and degradation, p65 phosphorylation, p38 phosphorylation, and ERK phosphorylation (59). Conversely, Depletion of endogenous TRAF3 enhances Act1 binding to the IL-17RA and promotes NFkB and MAPK activation as well as enhances IL-17A-dependent pro-inflammatory gene expression (59). TRAF3 suppresses the development of experimental autoimmune encephalomyelitis (EAE) and IL-17 mediated EAE in part by its role as a negative regulator of the IL-17 induced inflammation and autoimmunity.

### TRAF4

TRAF4, through its interaction with the adaptor protein Act1, was implicated in an intriguing pathway involving skin biology and tumor formation in squamous cell carcinoma (SCC) (60). IL-17 signaling is required TRAF4 for Act1-MEKK3 dependent extracellular signal-regulated kinase 5 (ERK5) activation, a pathway that is hyperactivated and dominant in human SCC. Through a positive feedback mechanism, IL-17-Act1-TRAF4-MEKK3-ERK5 induced the expression of target genes Steap4 (a metalloreductase for cell metabolism and proliferation) and p63 (a transcription factor for epidermal stem cell proliferation and Traf4 promoter). Thus, TRAF4 perpetuates its own expression through Steap4 and p63 and mediates IL-17 signaling in an Act1-MEKK3-ERK5 fashion contributing to hyperactivation of IL-17 inflammation and tumorigenesis. Curiously, the activation of MEKK3-ERK5 axis requires tyrosine phosphorylation. The tyrosine kinase responsible for IL-17-induced activation of the MEKK3-ERK5 axis was found to be EGFR. IL-17 stimulation prompts TRAF4 to recruit EGFR to the IL-17 receptor complex, while Act1 engages the kinase Src to phosphorylate the recruited EGFR, leading to its transactivation and the subsequent activation of MEKK3-MEK5-MEKK3 axis. Importantly, the IL-17-induced EGFR-ERK5 activation plays a crucial role in the expansion and migration of Lrig1+ cells and their progeny in the skin, critically contributing to wound healing and tumor formation. Of note, Lrig1 is a negative regulator of EGF-indued EGFR activation. Thus, the cis-activation of EGFR is impeded in the Lrig1+ stem cells residing in the hair follicle. Nevertheless, the Lrig1+ stem cells highly express TRAF4, which enables IL-17-induced trans-activation of EGFR, providing a mechanistic link between inflammation, wound healing and tumorigenesis (61).

Evidently, despite the role of TRAF4 in propagating and promoting IL-17-Act1 MEKK3-ERK5 pathway and its implication in squamous cell carcinoma, TRAF4 is also involved in negative regulation of the IL-17 cascade. TRAF4 deficiency leads to elevated basal as well as IL-17-induced levels of ERK1/2, JNK activation, degradation of IkBα, and gene expression CXCL1, G-CSF, and GM-CSF. Conversely, increasing TRAF4 expression leads to attenuation of the above mentioned IL-17 effects. Noteworthy was that TRAF4 deficiency *in vivo* led to marked exacerbations of the murine model of EAE (62). Thus, TRAF4 can be a potent positive as well as negative regulator of the IL-17 pathway. The mechanism of this negative regulation of TRAF4 involves the competition for the two TRAF binding site (TB) on Act1 between TRAF4 and TRAF6. This was demonstrated by the fact that TRAF6 association with Act1 was increased in TRAF4 deficiency; TRAF6 association with Act1 was reduced with TRAF4 overexpression; and the demonstrated that mutation of the TB site abolished TRAF4 interaction with Act1.

TRAF4 on the other hand seems to play a crucial role in IL-25-mediated type 2 immunity. TRAF4 in the T cell compartment and the epithelial compartment were shown to be required for the induction of IL-25 dependent genes, as well as, IL-25 mediated type 2 helper responses (Th2) *in vitro* as well as *in vivo* (63). While TRAF3 was not required for IL-25 induced responses, TRAF4 deficiency rendered an abolishment of IL-25 induced Cxcl1, Ccl11, IL-9, IL-13, IL-25 gene expression, IL-25 induced pulmonary responsiveness, as well as allergic airway inflammation. While it was previously shown that Smurf2 (smad ubiquitin regulatory factor 2) an E3 ubiquitin ligase, can target the proteasomal degradation of DAZAP2 (DAZ associated protein 2), an inhibitory molecule that interacts with the cytoplasmic domain of IL-17RB (64), blocking ACT1/IL-17RB interaction. The details of Smurf2 interaction were demonstrated to involve TRAF4. TRAF4 is shown to mediate K48 polyubiquitination and proteasomal degradation in order to allow for subsequent interaction of Act1 with the IL-25 receptor complex. Thus, TRAF4-mediated silencing of Dazap2 increased ACT1/IL-25R interaction and IL-25 responsiveness. Thus, TRAF4 requirement for IL-25 signaling is mediated in part by its mediation of the proteasomal degradation of Smurf2, and consequently, the removal of its inhibitory effect on the downstream signaling of the IL-25 receptor complex. Notably, IL-17 responsiveness in these studies was increased in TRAF4 deficiency consistent with the established role of TRAF4 as a negative regulator of the IL-17 pathway.

In summary, TRAF4 modulation of the IL-17 signaling pathway seems to be context and cell dependent. TRAF4 through Act1-MEKK3-ERK5 can lead to hyperactivation of IL-17 effects, while TRAF4 overexpression attenuates IL-17 effects. TRAF4 deficiency exacerbates IL-17 mediated inflammation in EAE model, but rendered resistance to IL17E (IL-25) induced effects.

## Concluding Remarks

Over the past 20 years, significant advancements has been made in the understanding of how Tumor necrosis factor receptor (TNFR)-associated factors (TRAFs) impact signaling pathways of IL-17 cytokines and drive protective immunity and mediate pathology. TRAFs are necessary in the activation as well as resolution of IL-17 signaling pathways and it is apparent that their dysregulation is implicit in disease pathobiology. Little is known about the involvement of TRAF1 and TRAF7 in the IL-17 signaling cascade and limited studies have explored the role of TRAFs in other IL-17 cytokine family members. Better understanding of the dynamics and mechanisms of how TRAFs regulate these inflammatory pathways are prudent for the development of new generation of therapeutics for the treatment of chronic inflammation, autoimmunity, and cancer.

## Author Contributions

All authors listed have made a substantial, direct and intellectual contribution to the work, and approved it for publication.

### Conflict of Interest Statement

The authors declare that the research was conducted in the absence of any commercial or financial relationships that could be construed as a potential conflict of interest.
